# Exploring japonica rice epigenetic diversity in the main production regions of Heilongjiang Province

**DOI:** 10.1038/s41598-022-08683-2

**Published:** 2022-03-17

**Authors:** Guifang Zhang, Nuo Li, Dongjie Zhang, Zhijiang Li, Aiwu Zhang, Xijuan Guo

**Affiliations:** 1 National Coarse Cereal Engineering Technology Research Center, Heilongjiang Bayi Agricultural University, Daqing, 163319 Heilongjiang People’s Republic of China; 2 College of Food Science, Heilongjiang Bayi Agricultural University, Daqing, 163319 Heilongjiang People’s Republic of China

**Keywords:** Plant sciences, Epigenetics analysis

## Abstract

As a major epigenetic modification, DNA methylation plays an important role in coordinating plant responses to environmental changes. Methylation-sensitive amplified polymorphism (MSAP) technology was used in this study to investigate the epigenetic diversity of fifty japonica rice samples from five regions in Heilongjiang Province, China. In addition, the phenotypic indicators of japonica rice samples and the environmental conditions of the sampling sites were investigated and analysed. Based on the MSAP analysis technique, using eight pairs of selective primers, we identified a total of 551 amplified loci, of which 267 (48.5%) were classified as methylation loci. The methylation status and levels of the japonica rice genome in different regions differed significantly (p < 0.05). The results of the analysis of molecular variance (AMOVA) revealed that most of the molecular variation (91%) came from within the groups (regions) and was caused by individual variation within the region. Furthermore, the results of principal coordinates analysis (PCoA), cluster analysis, and population structure analysis indicated that there was no obvious correlation between the epigenetic differences and geographical locations, which may have been due to the limited range of sampling sites. When environmental factors, phenotypic indicators, and epigenetic data analysis are combined, it is easy to conclude that japonica rice grown in the same latitudinal region has increased epigenetic and phenotypic similarities due to similar climatic conditions and production practices.

## Introduction

Approximately half of the world's population eats rice as a staple food^1^. As the world's leading rice producing region, Asia is home to many of the world's top rice exporting countries^2^. As one of the main production areas of high-quality japonica rice in China, Heilongjiang Province is the coldest rice cultivation area in the world^3^. The rice planting area here accounts for approximately 12.8% of the total rice planting area in China^4^, the commodity volume of rice accounts for 34.2% of that in the country, and japonica rice accounts for 68.6% of production in the country. Heilongjiang Province has a cold environment with long sunshine hours, a large day-night temperature difference, and fertile soils, with high organic matter contents^5^. These unique geographical advantages ensure the unique high quality of Heilongjiang japonica rice. Rice is rich in protein, fat, starch and other nutrients, and its quality is affected by many factors, such as variety, genetics, geographical environment, growth conditions and processing.

The biological environment and geographical differences can affect genetic diversity. To adapt to different environmental and developmental conditions, environmental factors and developmental signals will induce certain adjustments in genes, leading to changes in the final phenotype^6^. Many previous studies have focused on the environmental heterogeneity and wide distribution of varieties, exploring the impact of different geographical distances and environmental factors on genetic diversity^7–9^. Few studies, however, attempted to explain the influence of environmental and geographic factors on phenotypic polymorphism by epigenetic factors. Epigenetics is a branch of genetics that investigates heritable changes in gene expression do not arise from the nucleotide sequences of the genes. DNA methylation, genomic imprinting, maternal effects, gene silencing, nucleolar dominance, dormant transposons activation, RNA editing, and other epigenetic phenomena exist^10,11^. DNA methylation, unlike DNA mutations, is a key epigenetic modification that influences gene regulation in response to environmental signals and also has evolutionary importance for adaption to various environments across extended evolutionary time periods^12^. DNA methylation variation in rice can be caused by diseases^13^, droughts^14,15^, anaerobic conditions^16^, radiation^17^, high salinity^18^, heavy metals^19^, nutritional deficiency^20^ and other biotic and abiotic environmental stresses. According to growing evidence, epigenetic mechanisms play a vital role in coordinating the heritable and reversible changes in plant gene expression.

The methylation-sensitive amplified polymorphism (MSAP) technique, as a molecular technology that is a modification of amplified fragment length polymorphism (AFLP) techniques^21^, has been widely employed to analyse genomic DNA methylation levels and patterns in many species due to its dependability, high sensitivity, and convenience. It is possible to determine the methylation status of hundreds of anonymous loci distributed throughout the genome without requiring a reference genome^22^. MSAP is dependent on two different DNA methylation sensitive restriction isoschizomers, *Hpa II* and *Msp I*, which recognize the same tetranucleotide restriction site (5’-CCGG-3’), but *Hpa II* is sensitive to full methylation and cleaves the hemimethylated external cytosine, while *Msp I* is sensitive to only external cytosine methylation of the restriction site^23^. Previous studies^15,24,25^have proven that the MSAP technique is efficient for verifying the relationship between environmental factors and epigenetic diversity. Therefore, with the MASP molecular marker, this study aims to systematically assess the epigenetic diversity within and between Japonica rice from the five main production regions of Heilongjiang Province; the diversity is represented by genome-wide DNA methylation levels, patterns, and population structures. Exploring the phenotypic characteristics of landraces in different regions as well as elucidating the epigenetic diversity of rice in various growth environments and geographical locations, is important for preserving locally adapted varieties, determining basic agronomic characteristics of landraces, and improving rice quality and yield.

## Results

### Environmental conditions among the regions

The environmental data for each of the five regions of Heilongjiang Province were collated from China’s daily surface climate dataset of the China Meteorological Administration (http://www.cma.gov.cn). Monthly (line chart) and annual (column chart) average data for precipitation, relative humidity, sunshine hours, temperature, atmospheric pressure, and wind speed from five different rice production regions are presented in Figure S1. According to monthly data from various regions, the high temperatures and precipitation in each region were primarily concentrated from June to September. During this time, the humidity in each region increased significantly, while the sunshine hours decreased, and the atmospheric pressure also slightly decreased. Windy weather is most common from March to May in each region. From the annual average data of each region, it could be seen that except for the atmospheric pressure and wind speed, which showed significant differences in each of the five regions, the other four indicators showed no significant differences. The annual average atmospheric pressures ranged from 978 Pa (XS) to 1005 Pa (JSJ), with wind speeds ranging from 2.4 m/s (CHY) to 3.8 m/s (JSJ). Although the difference between regions was not statistically significant, the annual average precipitation values in the five regions were observed to be in the following order: WC (24 mm) > XS (23 mm) > FZ (22 mm) > JSJ (19 mm) > CHY (16 mm). The annual average sunshine hours, which were adversely connected to precipitation, ranged from 6.1 h/d (WC and XS) to 7.0 h/d (CHY). The annual average humidity in each region ranged from 63% (XS and CHY) to 68% (WC). The annual average temperature in each region ranged from 3.6 °C to 4.9 °C. In particular, the annual average temperature in the XS and WC regions was slightly higher than in the other three regions.

### Phenotypic data assays

The results of rice phenotype determination in the five regions are shown in Table S1, and the differences in rice phenotype data in each region were significant. The average 1000-grain weight varied from 25.73 g (JSJ) to 27.09 g (WC). Plant height ranged from 89.8 cm (CHY) to 99.5 cm (WC), while panicle length varied from 16.2 cm (JSJ) to 20.7 cm (WC). Brown rice length and brown rice shape ranged from 5.1 mm (JSJ) to 6.5 mm (WC) and 1.7 (JSJ) to 2.6 (WC), respectively. When all of the phenotypic data were combined, it could be found that, except for plant height, the phenotypic indicator values for rice in the JSJ region were the lowest, while those for the WC or XS regions were relatively high.

### MSAP for selective primer combinations

The eight pairs of MSAP selective primers were used to amplify genomic DNA from 50 samples from five distinct regions to assess the methylation status in Heilongjiang japonica rice. The total number of loci generated by a selective primer combination ranged from 51 (E83/M40) to 84 (E83/M42), with amplicon lengths ranging from 37 to 495 bp (Table 1). A total of 551 amplified loci and an average of 449 polymorphic loci were identified. The percentages of polymorphic loci in each selective primer-pair combination were all greater than 75% (74.8–93.4%), with the E83/M40 primer combination having the highest percentage (93.4%). Of the total number of markers (551 loci), 267 (48.5%) were classified as methylation loci and 284 (51.5%) were classified as nonmethylation loci. The E85/M42 primer combination yielded the most methylation loci, while the E83/M40 primer combination yielded the fewest.

**Table 1:** Number of loci across eight pairs of selective primer combinations with respective methylation loci, nonmethylation loci and percentages of polymorphic loci.

Selective primer combination	Amplicon length (bp)	Number of amplified loci	Number of polymorphic loci	Percentages of polymorphic loci (%)	Number of methylation loci	Number of nonmethylation loci
E36/M42	40–469	74	59	79.8	38	36
E36/M50	37–435	68	61	90.2	27	41
E40/M40	41–464	68	55	81.4	30	38
E40/M50	40–430	67	57	85.6	27	40
E83/M40	41–367	51	48	93.4	24	32
E83/M42	42–495	84	67	79.7	44	40
E83/M59	38–481	65	49	75.9	35	30
E85/M42	39–308	74	55	74.8	47	27
Total	–	551	449	81.5	267	284

### Analysis of DNA methylation levels in different of rice production regions

To compare the methylation levels of five groups, ANOVA and Duncan's multiple comparison were used. There were significant differences in DNA methylation levels among the 551 raw MSAP loci generated by 50 rice samples, which were artificially divided into five groups based on region (Table 2). The percentages of Type I in five groups were from 48.82% (JSJ) to 54.77% (WC), Type II ranged from 15.23% (FZ) to 17.01% (CHY), Type III ranged from 12.92% (CHY) to 15.79% (JSJ) and Type IV ranged from 15.81% (WC) to 20.33% (FZ). There were no statistically significant differences in total methylation levels among the five groups, but there were significant differences in full methylation, hemi-methylation, and nonmethylation levels (p < 0.05). The total methylation levels of rice in regions FZ, CHY, JSJ, XS and WC ranged from 48.03% to 51.18%; the corresponding full methylation levels ranged from 31.62% (WC) to 36.19%, hemi-methylation levels ranged from 12.92% (CHY) to 15.79%, and nonmethylation levels ranged from 48.82% (JSJ) to 54.77% (WC). The regions with the lowest levels of full methylation, hemi-methylation, and nonmethylation were WC, CHY, and JSJ, respectively.

**Table 2:** Analysis of DNA methylation levels in different regions of rice.

Region	Type I (%)	Type II (%)	Type III (%)	Type IV (%)	Total methylation level^1^(%)	Full methylation level^2^(%)	Hemi-methylation level^3^(%)	Nonmethylation level^4^(%)
FZ	50.96^ab^	15.23^a^	13.48^ab^	20.33^c^	49.04^a^	35.55^bc^	13.48^ab^	50.96^ab^
CHY	50.89^ab^	17.01^a^	12.92^a^	19.18^bc^	49.11^a^	36.19^c^	12.92^a^	50.89^ab^
JSJ	48.82^a^	15.57^a^	15.79^b^	19.82^c^	51.18^a^	35.39^bc^	15.79^b^	48.82^a^
XS	51.96^ab^	15.57^a^	15.39^ab^	17.08^ab^	48.04^a^	32.65^ab^	15.39^ab^	51.96^ab^
WC	54.77^b^	15.81^a^	13.61^ab^	15.81^a^	48.03^a^	31.62^a^	13.61^ab^	54.77^b^

a–c different lowercase letters represent significant different among the five regions, p < 0.05; 1. Total methylation level: (Type II + Type III + Type IV)/(Type I + Type II + Type III + Type IV) × 100%; 2. Full methylation level: (Type III + Type IV)/(Type I + Type II + Type III + Type IV) × 100%; 3. Hemi-methylation level: (Type II)/(Type I + Type II + Type III + Type IV) × 100%; 4. Nonmethylation level: (Type I)/(Type I + Type II + Type III + Type IV) × 100%.

### Analysis of epigenetic diversity in different regions

These 551 informative loci further yielded 2204 polymorphic subepiloci in five different regions (Table 3). GENALEX software (version 6.5, http://biology.anu.edu.au/GenAlEx) was used to calculate the percentage of polymorphic loci (*PLP*%), the number of effective alleles (*Ne*), Shannon's information index (*I*), and Nei's gene diversity (*h*). The percentages of polymorphic loci (*PLP*%) for the five groups ranged from 49.09% (CHY) to 52.63% (FZ), with an average of more than 50%, indicating that the five populations were highly polymorphic. Compared with other groups, all indices (including *Ne*, *I* and *h)* related to polymorphism were highest in XS (1.292, 0.266, and 0.175), and lowest in CHY (1.261, 0.245, and 0.160). Using the final epigenetic data matrix obtained by transforming the four recognized methylation types of each multistate epilocus into independent binary subepiloci, AMOVA was performed to quantify epigenetic variation among five regions. The majority of epigenetic variance (91%) occurred within the groups, whereas the differentiation index (*Φ*st) of 8.5% indicated moderate epigenetic differentiation between them (Table 4).

**Table 3:** Epigenetic diversity index values of rice in different regions.

Region	No. of samples (N)	No. of loci	Percentage of polymorphic loci (*PLP* %)	Average no. of effective alleles (*Ne*)	Shannon’s information index (*I*)	Nei’s gene diversity (*h*)
FZ	10	2204	52.63	1.284	0.264	0.173
CHY	10	2204	49.09	1.261	0.245	0.160
JSJ	10	2204	50.77	1.283	0.260	0.171
XS	10	2204	51.59	1.292	0.266	0.175
WC	10	2204	49.73	1.268	0.249	0.163

*Ne* Number of effective alleles, *I* Shannon’s information index, *h* Nei’s gene diversity.

**Table 4:** Analysis of molecular variance (AMOVA) of rice in different regions.

Source of variation	Degrees of freedom(df)	Sum of squares (SS)	Estimates of variance components (Est. var.)	Percentage of variation (%)	*Φ*st	*p* value (rand ≥ data)^1^
Among groups	4	1592.400	19.197	9%	0.085	0.0001
Within groups	45	9275.800	206.129	91%		
Total	49	10,868.200	225.326	100%		

Probability of a random value greater than or equal to the observed data value based on permutation test (*n* = 9999).

The results of Nei’s genetic identity and genetic distance analysis showed a generally high level of genetic identity as well as a low level of genetic distance (Table 5). The genetic distance between FZ and CHY was the lowest (0.030) and the genetic identity was the highest (0.970) among the five different regions, showing that the FZ and CHY populations were the most closely related. The highest genetic distance value (0.063) and the lowest genetic identity value (0.939) occurred in WC and CHY, indicating the farthest genetic relationship between them.

**Table 5:** Analysis of genetic identity (below diagonal) and genetic distance (above diagonal) among rice in different regions based on Nei’s genetic distance.

Region	FZ	CHY	JSJ	XS	WC
FZ	–	0.030	0.037	0.042	0.056
CHY	0.970	–	0.031	0.045	0.063
JSJ	0.963	0.970	–	0.040	0.059
XS	0.958	0.956	0.961	–	0.039
WC	0.945	0.939	0.943	0.961	–

### Principal coordinate analysis of different rice production regions

**Figure 1:**
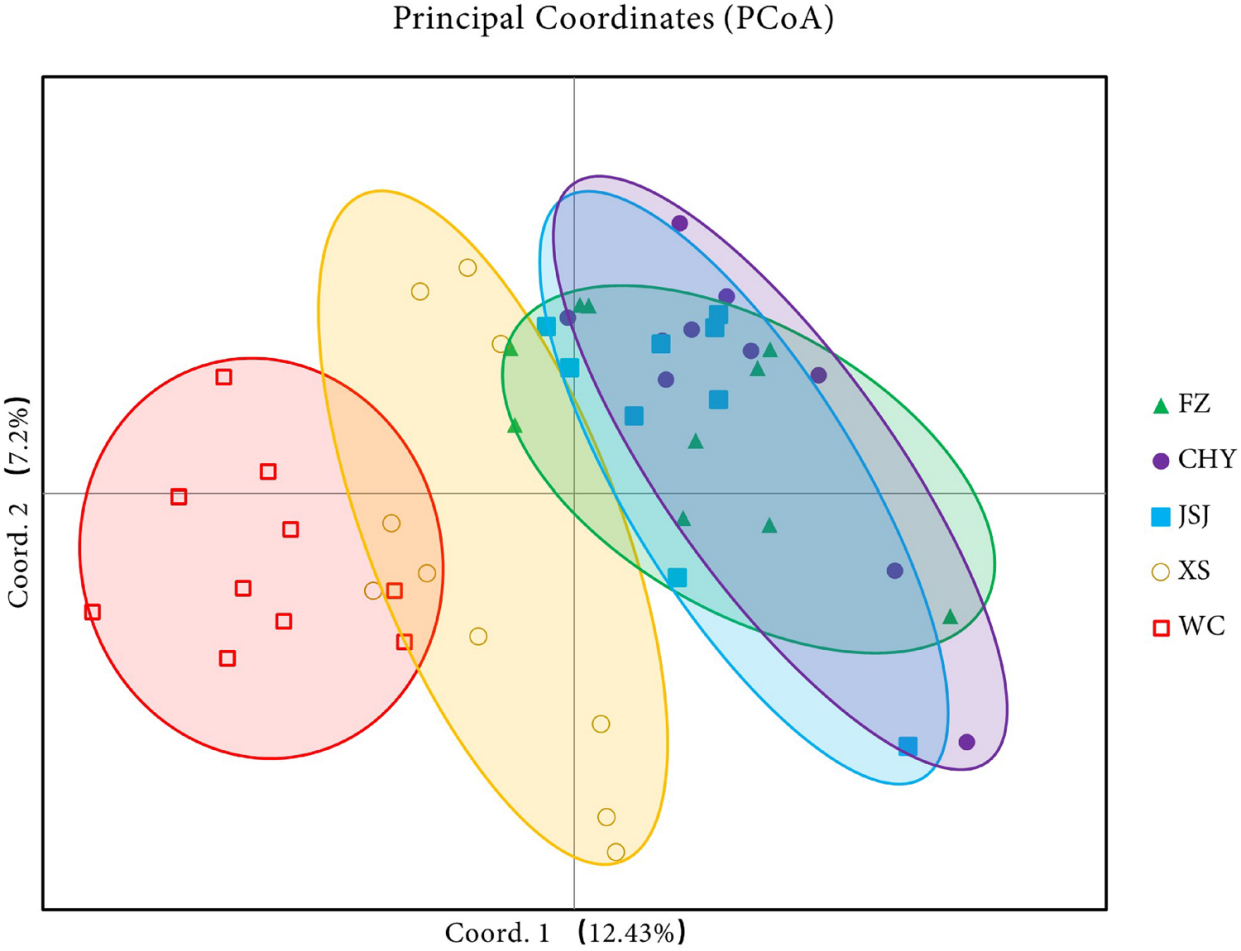
Principal coordinate analysis (PCoA) based on the genetic distance matrix of the total subepiloci matrix for individuals from different regions.

The epigenetic structure of five different rice production regions was estimated using principal coordinate analysis (PCoA) based on genetic distance among the 50 sampled individuals (Fig. 1). Although the first two principal coordinates accounted for 19.63% of the genetic variation of the rice population in the PCoA plot, indicating a scattered distribution of five regions, the main contributor (12.43%) to the detected variability was associated with the distribution on the east–west axis, where the populations WC and XS in the west were close and the populations CHY, JSJ, and FZ in the east were close. The PCoA result indicating the greatest distance between WC and CHY was consistent with the results of the full methylation level, genetic identity, and genetic distance of rice in different regions.

### Cluster analysis

**Figure 2:**
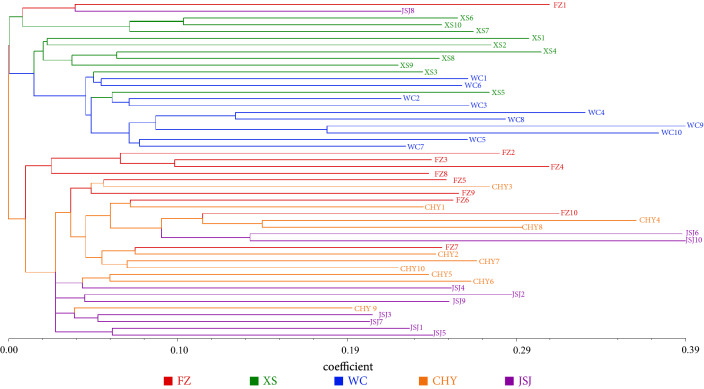
Neighbour-joining (NJ) phylogenetic tree of 50 rice from five different regions.

The NJ phylogenetic tree based on the genetic distance of four types of subepiloci binary matrix data showed that the 50 individuals from five different regions were divided into two clades (Fig. 2). Populations WC and XS were in the same clade, whereas populations FZ, CHY, and JSJ were divided into a second clade, which coincided with the PCoA results. Although each clade was shared by 2–3 populations, indicating that the population distribution did not exhibit distinct genealogical geographic patterns, most of the individual samples were clustered according to their regions.

### Analysis of epigenetic population structure

**Figure 3:**
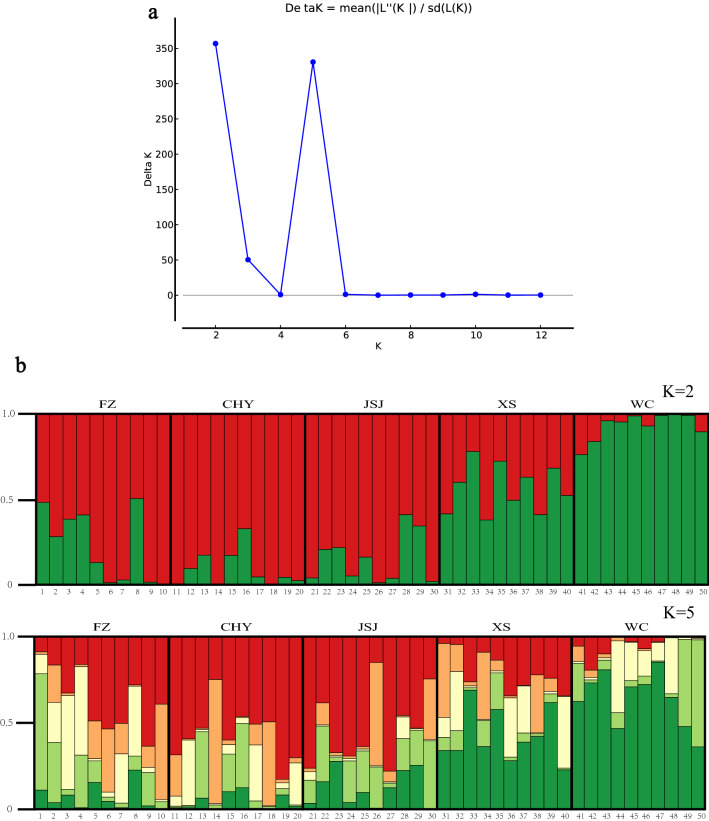
Epigenetic population structure of the 50 rice individuals based on the subepiloci binary matrix data transformed from MSAP dataset. (**a**) Inference of the optimal number of subpopulations using the delta *K* variation (∆*K*) with *K* varying from 1 to 13. (**b**) Bar plot with each column representing the estimated membership coefficients for each individual sample, which were represented with numbers from 1 to 50 and grouped into FZ, CHY, JSJ, XS and WC. The proportion of the colour making up each column represents the proportion contributed by the subpopulation. Subpopulations 1 and 2 are represented by red and green colours, respectively (*K* = 2); Subpopulations 1, 2, 3, 4 and 5 were represented by red, orange, yellow, light green, and green colors, respectively (*K* = 5).

The analysis of epigenetic population structure using subepiloci binary matrix data transformed from the MSAP dataset of 50 samples revealed that the ∆*K* criterion reached its nodal value when *K* = 2 (Fig. 3a, *K* = 2, ∆*K* = 356.9; *K* = 5, ∆*K* = 330.7), indicating that there were two distinct subpopulations at the uppermost level of the epigenetic structure. The results for *K* = 2 and *K* = 5 were presented to illustrate the formation of populations (Fig. 3b). When *K* = 2, the entire population was split into two subpopulations. Subpopulation I (red colour) was primarily present in the CHY, FZ, and JSJ populations, whereas most individuals in population WC were assigned to subpopulation II (green color), except for the population XS, which was assigned into both subpopulation I (0.56) and subpopulation II (0.43). When *K* = 5, subpopulation II was further subdivided into three clusters (green, light green, and yellow), while light green and yellow clusters were found in some individuals from population FZ.

## Discussion

Previous research on rice has generally studied the genetic diversity of varieties and accessions using molecular marker techniques such as SSR (simple sequence repeat) and SNPs (single nucleotide polymorphism), and the impact of geographical environment and varieties on population structure has been analysed and clarified^7,8,26,27^. Apart from genetic mechanisms, increasing evidence has shown that epigenetic processes have important evolutionary significance in plant adaptation to stressful environments^12,28^. Among these epigenetic mechanisms, cytosine DNA methylation has received the most attention and has provided the most examples of transgenerational stability^29^. For example, mangrove plants from different nearby habitats with similar genetic profiles have divergent epigenetic profiles, indicating that cytosine methylation variation can be more closely correlated with environmental variation than genetic variation^30^. The results of global DNA methylation patterns of grapevines from different regions revealed that epigenetic profiles differed significantly despite the low genetic differentiation between vineyards^31^. In recent years, MSAP, as a modified AFLP technique, has been widely used to detect the genomic methylation status and extent of diverse species^24,32^.

Many extrinsic and intrinsic factors affect the growth and development of plants during the vegetative and reproductive phases of the lifecycle and thus affect grain yield^33^. Meteorological conditions such as temperature, precipitation, and sunshine undoubtedly cause epigenetic effects, which then cause phenotypic variation^34^, and will eventually affect the yield and quality of rice^35^. In this study, six environmental factors that significantly affect the growth and development of rice and five phenotypic indicators that reflect rice quality were selected to discuss the effect of environmental conditions on phenotypic and epigenetic diversity. The results of the environmental condition analysis of five geographically isolated regions showed that most environmental data had no significant difference, which may have been due to the selected regions being all located in Heilongjiang Province, so the climate conditions were relatively similar. Even so, similar environmental conditions still generate significant phenotypic diversities (shown in Table S1); for this reason, we must ask what factors influenced the phenotypic differentiation among these regions. By comparing the annual average meteorological data of different regions, we found that although the data differences were not statistically significant, the Wuchang and Xiangshui regions had relatively higher precipitation, humidity, and temperature, as well as lower atmospheric pressure and wind speed than the other three regions. Based on the phenotypic data, we can speculate that such abundant phenotypic variation was influenced by the combined action and interaction of many factors, such as temperature, humidity, and sunshine. These distinct and complex environmental conditions contribute to the specific quality of rice produced in these regions.

In this work, eight pairs of polymorphic primers were selected to analyse the genome-wide methylation status of 50 japonica rice from five geographically regions of Heilongjiang Province. There were 551 amplified loci in total, with the percentages of polymorphic loci in each selective primer combinations all greater than 75%, indicating that this rice variety has relatively high polymorphism compared to other rice varieties^36^. Furthermore, we found that 48.5% of 551 CCGG sites in the genome were methylated, but the proportion of cytosine methylation varied depending on the selective primer combination, indicating that their ability to capture genomic areas susceptible to methylation varied.

When we compared the methylation patterns of the 50 rice samples from various regions, we discovered a significant difference in both methylation type and methylation level. Compared with other groups, the genome methylation level of rice from the Wuchang region was the lowest, regardless of internal or external cytosine methylation. The internal cytosine methylation level of rice from the Chahayang region was the lowest, the external cytosine methylation level of rice from Jiansanjiang was the lowest, and the difference in methylation level between rice from the Chahayang region and Wuchang region was the most significant. We also obtained consistent results when analysing genetic identity and genetic distance. Nei's genetic distance refers to "the degree of genetic differences between different populations or species, and a certain value was used to measure the scale of genetic differences between populations or species"^37^. Population genetic structure and genetic differentiation degree can be inferred by calculating Nei’s genetic distance. The value of genetic distance also reflected the genetic relationships of various groups and was an important indicator of population diversity. The results of genetic identity and genetic distance analyses showed that the genetic relationships between rice from Fangzheng and Chahayang were the closest, while the genetic relationship between Wuchang and Chahayang was the farthest. These results revealed a population-specific epigenetic pattern based on geographical regions.

Based on the results of AMOVA, it was observed that a wide range (91%) of within group(region) differences were caused by individual variation within regions. Such high levels of molecular differences between individuals in the same region could be explained by the random accumulation of variation with age, which can be genetic or epigenetic in character. The apparent genetic variation of a single individual is a prerequisite for microevolution of plants^38^. The results of epigenetic diversity analysis showed that epigenetic diversity within regions was moderate (*PLP*%: 49.09% ~ 52.63%, *I*: 0.245 ~ 0.264, *h*: 0.160 ~ 0.175) but did vary across regions. The polymorphism index of rice in the Chahayang and Wuchang regions was relatively low, and that in the Fangzheng and Xiangshui regions was relatively high. Understanding the causes and consequences of natural epigenetic variation is difficult because it can be influenced by a variety of factors^39^. The difference in epigenetic diversity among different regions might be related to the niche variation hypothesis^40^; that is, if the habitat range of the population was wider and more diverse, there would be more variation than if the population had a narrow habitat range.

Although some individuals were clustered in other regions, the majority of individuals from each region were clustered in the same clade, possibly indicating that individuals within the same region have epigenetic similarity due to a relatively consistent geographical environment, climatic conditions, soil composition, production practices, and other factors (Fig. 2). The PCoA results showed that the main variation was associated with the distribution on the east–west axis, that the Wuchang and Xiangshui regions were clustered together in the west part, and that others were clustered together in the east part (Fig. 1). In addition, the epigenetic structure of the japonica rice genotypes also revealed two distinct subpopulations. Consistency between the cluster grouping on the phylogenetic tree (Fig. 2) and the subpopulation determined by structure analysis was also observed (Fig. 3). Subpopulation 1 (red colour) consisted primarily of the cluster groups of Fangzheng, Chahayang, and Jiansanjiang, whereas subpopulation 2 (green colour) consisted primarily of the cluster groups of Wuchang and Xiangshui. Observing the distribution of the five regions on the map of Heilongjiang, we found that the Wuchang (latitude range 44˚45′–44˚91′) and Xiangshui (latitude range 44°07′–44°15′) regions were relatively close to the south of Heilongjiang, the Chahayang (latitude range 48°17′–48°37′) and Jiansanjiang (latitude range 47°26′–48°00′) were distributed on the east and west sides of Heilongjiang respectively, which were more northwards than the Wuchang and Xiangshui regions, and the Fangzheng (latitude range 45°76′–45°88′) region was located between the north and the south. Therefore, comprehensive analysis of the PCoA, cluster and structure results in this study indicate that the epigenetic differences in japonica rice from different regions were not obviously associated with regional differences, but as the geographical latitude moved from south to north, the genetic relationship changed from close to far.

Interestingly, when we combined the analysis results of environmental conditions, phenotypic diversity and epigenetic diversity, we found that the phenotypic diversity results were closely related to the epigenetic diversity results. Compared with the Jiansanjiang region, rice in the Wuchang and Xiangshui regions had lower methylation levels, closer genetic distances, similar phenotypic data, and similar latitudes, altitudes and climatic conditions. Therefore, under long-term natural environmental selection and directional selection, to adapt to different environmental and developmental conditions, environmental factors and developmental signals will induce certain adjustments in plant genes, resulting in changes in the final phenotype.

## Conclusions

In this research, the epigenetic diversity of fifty rice samples from five different regions of Heilongjiang Province was investigated using eight pairs of MSAP primers. The differences in global genome methylation patterns and levels in rice of different regions were explored. AMOVA revealed that most of the genetic variation came from within populations. At the same time, the phenotypic analysis results of rice in different regions were consistent with the epigenetic analysis results. Due to the limited geographical scope of sampling, most environmental conditions in different regions did not differ significantly, and no obvious correlation between the epigenetic differences and geographic location was found, but it is not hard to speculate that they may have higher epigenetic and phenotypic similarity at the same latitude.

## Materials and methods

### Plant materials and experimental sampling design

**Figure 4:**
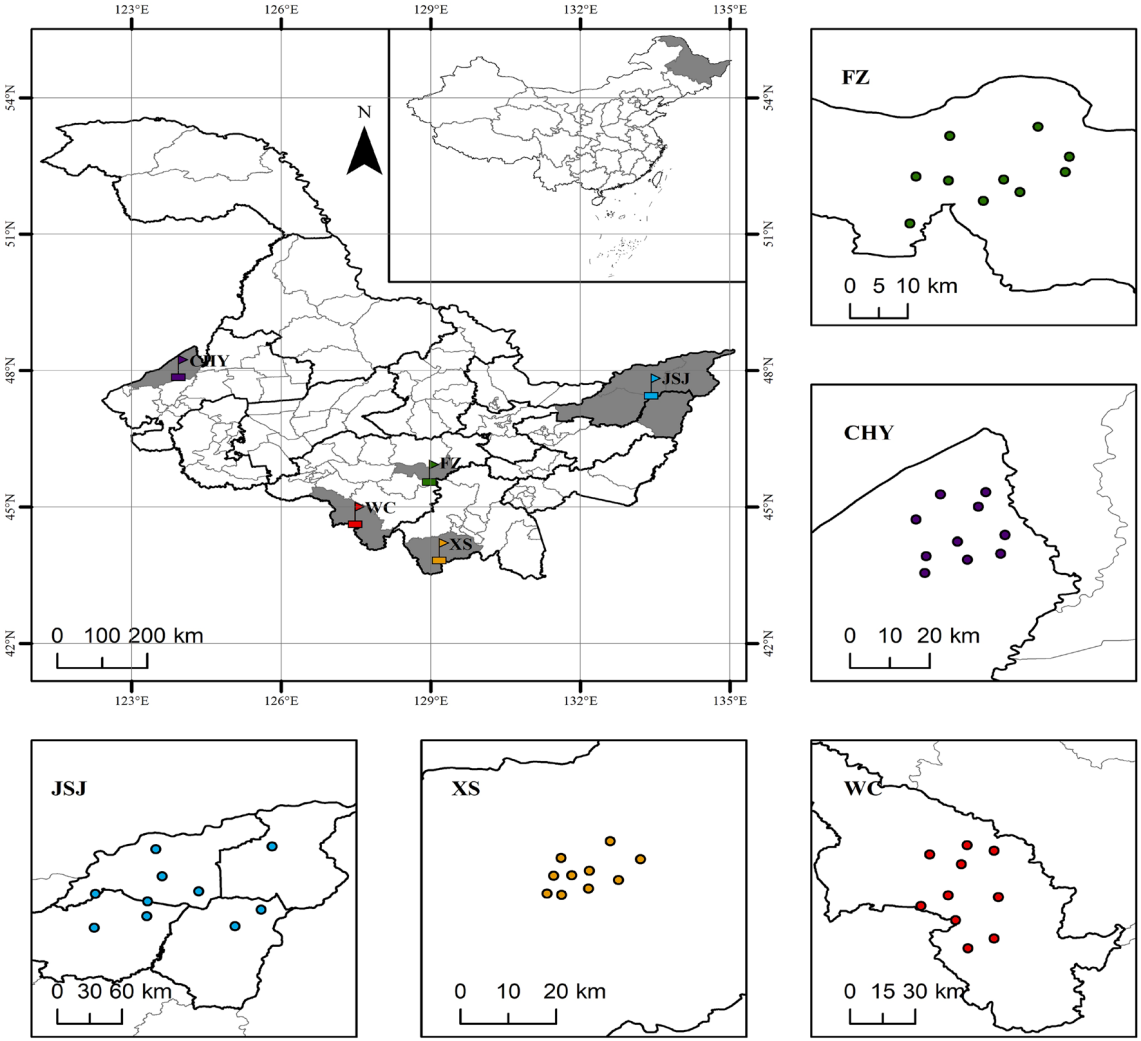
Distribution japonica sampling sites in Heilongjiang Province. The distribution map was generated by ArcGIS Pro software (https://www.esri.com/en-us/arcgis/products/arcgis-pro.) and was based on the latitude and longitude data measured at the sampling sites.

The purpose of sampling was to obtain representative samples to evaluate the composition or characteristics of the sample system. By investigating the geography, ecology, environment, accumulated temperature, climate, variety, production mode, and degree of mechanization in various regions of Heilongjiang Province, in this study, a total of 50 japonica rice varieties from five main farming regions in Heilongjiang Province were selected to investigate epigenetic diversity, including Fangzheng (FZ) and Wuchang (WC) in the central region, Chahayang (CHY) in the western region, Jiansanjiang (JSJ) in the eastern region, and Xiangshui (XS) in the southern region (Fig. 4). All japonica samples were local cultivated varieties and obtained from local fields in each region. In each region, 10 sampling fields were selected according to the principle of widest area coverage, and the collection time was set after the rice maturity period and before harvest. To ensure authenticity and representativeness, each field adopted a five-point sampling method to collect rice samples. The sampling amount of each field rice ear was not less than 2.0 kg. The sampling region and its basic geographic information are shown in Table 6. Following collection, the sample was dried in a ventilated and cool environment to a moisture content of 10–14%, then threshed and stored in a dark place before use.

**Table 6:** Japonica sampling regions and their basic geographic information.

Region	Group	Plant Number	Accumulative temperate zone	Average altitude	Longitude range	Latitude range
Fangzheng	FZ	10	Second	142 m	128°67′–128°86′	45°76′–45°88′
Chahayang	CHY	10	Second	311 m	124°09′–124°30′	48°17′–48°37′
Jiansanjiang	JSJ	10	Third	58 m	132°65′–134°13′	47°26′–48°00′
Xiangshui	XS	10	Second	254 m	129°10′–129°27′	44°07′–44°15′
Wuchang	WC	10	Second	450 m	127°43′–127°74′	44°45′–44°91′

### Environmental conditions

The site-specific environmental data were collected from publicly accessible websites based on the latitude and longitude coordinates of the sampling sites. The data was collated from China’s daily surface climate data of the China Meteorological Administration (http://www.cma.gov.cn). The differences between groups were analysed using one-way ANOVA with IBM SPSS software, and p < 0.05 indicated a significant difference.

### Phenotypic data

The phenotypic data were analysed using the Standard Evaluation System for Rice (SES)^34^, which was slightly modified (http://www.knowledgebank.irri.org/images/docs/rice-standard-evaluation-system.pdf). The five evaluation standard indicators at mature stages were selected to analyse the phenotypic diversity of rice in different regions, since the samples were all from mature rice landraces. The phenotypes were collected from ten plants or seeds from each sample. For the 1000-grain weight, grains harvested from ten plants were mixed, 100 randomly selected grains were weighed in grams with three replicates, and the average was multiplied by ten. To assess the phenotypic diversity of rice in the five regions, researchers utilized ANOVA and Duncan's multiple comparison.

### Isolation of total genomic DNA

Ten healthy japonica seeds of each sample were randomly selected and cultivated at a room temperature of 25°. The fresh young leaves of the 3-leaf stage seedlings were mixed together, and the genomic DNA was extracted from 100 mg of each sample by the improved CTAB method^41,42^ The sample was evenly mixed with 500 µL of CTAB buffer solution and 20 µL of proteinase K (20 mg/mL) evenly. After incubation at 65 °C for 60 min, 20 µL RNase A was added before centrifugation (1600 g for 10 min). With an equal volume of phenol and chloroform (1:1), the supernatant was extracted twice. The upper phase was combined with two-thirds volume of isopropanol. The mixture was centrifuged for 10 min. The pellet was washed with ethanol (70%, v/v) twice and centrifuged at room temperature for 10 min. The supernatant was discarded carefully, the washing was repeated once, and the pellet was dried. All genomic DNA samples were stored at − 20 °C for later use after being dissolved in sterile deionized water.

The concentration and purity of the extracted DNA were detected by a UV–VIS spectrophotometer (Merinton™ SMA4000, Merinton Instrument, Inc., USA) and 1.0% agarose gel electrophoresis. The OD 260/280 ratio was between 1.762 and 1.806, and the concentration was approximately 1000 ng/µL, indicating that the extracted DNA met the quality requirements for subsequent experiments^43^.

### MSAP analysis

Methylation-sensitive amplification polymorphism (MSAP) analysis is an improvement on the typical amplified fragment length polymorphism (AFLP) method, which is based on the differential sensitivity of a pair of isoschizomers (*Hpa* II and *Msp* I) to cytosine methylation^14,21^. In brief, DNA was double digested by *EcoR* I plus *Hpa* II and *EcoR* I plus *Msp* I (NEB, New England Biolabs (Beijing) LTD) in digestion reaction buffer. Digestion and ligation were performed simultaneously in 20 μL system which contained DNA (400 ng), T4 DNA ligase (5 units), *EcoR* I (5 units), *Hpa* II or *Msp* I (5 units), *EcoR* I adaptor (*EcoR* I adaptor I and *EcoR* I adaptor II; Table 7), *Hpa*/*Msp* adaptor (*Hpa/Msp* adaptor I and *Hpa/Msp* adaptor II; Table 3), 10 × T4 ligase buffer (2 μL) and sterile deionized water. The mixture was incubated for 12 h at 37 °C, and then the enzyme was inactivated after the reaction.

**Table 7:** Adaptors and primer sequences used for MSAP analysis.

	**Adaptor**	**Sequence(5’-3’)**
Adaptor	*EcoR* I adaptor I	CTCGTAGACTGCGTACC
	*EcoR* I adaptor II	AATTGGTACGCAGTCTAC
	*Hpa/Msp* adaptor I	GACGATGAGTCCTGAG
	*Hpa/Msp* adaptor II	CGCTCAGGACTCAT
Preamplification primer	*EcoR I* preamplification primer	GACTGCGTACCAATTC
	*Hpa/Msp* preamplification primer	GATGAGTCCTGAGCGG
Selective-amplification primer	E36	GACTGCGTACCAATTCACC
	E40	GACTGCGTACCAATTCAGC
	E50	GACTGCGTACCAATTCCAT
	E83	GACTGCGTACCAATTCTCA
	E85	GACTGCGTACCAATTCTCG
	M40	GATGAGTCCTGAGCGGAGC
	M42	GATGAGTCCTGAGCGGAGT
	M50	GATGAGTCCTGAGCGGCAT
	M59	GATGAGTCCTGAGCGGCTA

The optimal preamplification PCR system (20 μL) consisted of 10 mmol dNTPs (0.4 μL), 10 × buffer (2 μL), 5unit Taq polymerase (0.2 μL), and 10 μmol *EcoR* I preamplification primer (0.5 μL; Table 7), 10 μmol *Hpa/Msp* preamplification primer (0.5 μL; Table 7), 1.5 mmol magnesium chloride, and sterile deionized water. The thermal cycles for the PCRs were programmed as follows: predenaturation for 5 min at 94 °C, followed by 30 cycles of denaturing and annealing for 30 s at 94 °C, extending for 60 s at 72 °C, and a final extension for 10 min at 72 °C. The preamplification products were diluted 20-fold for selective amplification reactions.

A total of 5 samples were randomly selected from each region for the optimal primer selection test. The primers with a clear spectrum, strong signal and high polymorphism from the twenty different primer combinations (E36/M40, E36/M42, E36/M50, E36/M59, E40/M40, E40/M42, E40/M50, E40/M59, E50/M40, E50/M42, E50/M50, E50/M59, E83/M40, E83/M42, E83/M50, E83/M59, E85/M40, E85/M42, E85/M50, E85/M59, Table 3) were selected as the optimal selective amplification primers (E36/M42, E36/M50, E40/M40, E40/M50, E83/M40, E83/M42, E83/M59, E85/M42). The selective amplification PCR was conducted using the same method as the preamplification PCR^44^.

After mixing formamide and the molecular weight internal standard (ROX500) at a volume ratio of 100:1, the PCR products were diluted tenfold (1 µL) and mixed with the above mixture (9 µL) in each well of a 96-well sample plate. The PCR products were analysed on an ABI 3730XL auto DNA sequencer (Applied Biosystems™, USA). The selection of MSAP fragments was restricted to allele sizes ranging from 75 to 500 bp^45^.

### MSAP marker genotyping

The resulting chromatograms were visually displayed using GeneMarker software (version 2.2.0, https://softgenetics.com/GeneMarker.php) by detecting signals of varied intensities and locations^46^. The binary matrix comprising information on epilocus presence (1) or absence (0) was generated by side-by-side comparisons of the banding patterns of each *EcoR* I/*Hpa* II and *EcoR* I/*Msp* I enzyme combination. Four DNA methylation types were artificially categorized as follows: type I (1/1), type II (0/1), type III (1/0) and type IV (0/0). The modified approach of "mixed-scoring" was used to study the epigenetic diversity of rice in different regions to test the specific influence of different methylation types. The final epigenetic data matrix was generated by transforming each multistate epilocus and four recognized methylation types into binary subepiloci^47,48^(Table 8).

**Table 8:** Four DNA methylation types, their degrees and modified mixed scoring approach.

Types	Methylation status	Methylation status	Banding pattern (*EcoR* I/*Hpa* II)/(*EcoR* I/*Msp* I)	Methylation degree	Mixed scoring (Subepiloci)			
					I	II	III	IV
I	Non methylation	^5’^CCGG GGCC_5’_	1/1	0	1	0	0	0
II	Full and hemi-methylation of internal cytosine	^5’^C^m^CGG GGC^m^C_5_ ^5’^C^m^CGG G GCC_5’_	0/1	1	0	1	0	0
III	Hemi-methylation of external cytosine	^5’m^CCGG GGCC_5’_	1/0	1	0	0	1	0
IV	Full and hemi-methylation of both cytosines Mutation(unknown) Full methylation of external cytosine	^5’m^C^m^CGG GGC^m^C^m^_5’_ ^5’^CCTG GGNC_5’_ ^5’m^CCGG GGCC^m^_5’_	0/0	2	0	0	0	1

### Data analysis

The original binary matrix data was used to estimate the level of DNA methylation. One-way analysis of variance (ANOVA) and Duncan's multiple comparison were employed in IBM SPSS statistics software (version 24.0.0, https://www.ibm.com/cn-zh/products/spss-statistics) to analyse significant differences in methylation levels in different regions of rice. The epigenetic data matrix was analysed with GENALEX software (Version 6.5)^49^ to calculate diversity parameters, including the percentage of polymorphic loci (*PLP*%), number of effective alleles (*Ne*), Shannon’s information index (*I*) and Nei’s gene diversity (*h*). The analysis of molecular variance (AMOVA) method was applied to estimate population epigenetic differentiation within and between the five regional populations. Next, we used principal coordinate analysis (PCoA) to estimate epigenetic structure based on the genetic distance matrix of the total subepiloci matrix.

We assessed the population structure using the Bayesian clustering model implemented in STRUCTURE software (version 2.3.4, http://pritchardlab.stanford.edu/structure.html)^50^ based on four types of subepiloci binary matrix data (I, II, III, and IV). By clustering individual genotypes into a certain number of populations (*K*) and reducing divergence from Hardy–Weinberg equilibrium, this model infers population structure. We varied *K* value from 1 to 13, assumed 100,000 initial interactions (burn-in period) and 1,000,000 Monte Carlo Markov chain (MCMC) interactions, and conducted 10 independent iterations for each *K*. *K* was calculated using the method based on the likelihood's second-order rate of change (DK)^51^. Structure Harvester (version 0.6.94, http://taylor0.biology.ucla.edu/structureHarvester)^52^ was used to evaluate the results and determine the most likely value of *K*. Repeated sampling analysis was conducted on the results of structure analysis by CLUMPP software (version 1.1.2, http://rosenberglab.bioinformatics.med.umich.edu/clumpp.html)^53^, and the Q-matrix results for the best *K* value were obtained. A structure graph was drawn by DISTRUCT software (http://rosenberglab.bioinformatics.med.umich.edu/distruct.html) according to the results of repeated sampling analysis. The neighbor-joining cluster analysis method based on Nei's genetic distance data was performed using the NTSYS-pc software (version 2.0, http://www.exetersoftware.com/cat/update.html).

### Ethics statement

All the rice materials were obtained in the local fields of each region with the help of the local Bureau of Agriculture and Rural Affairs. We have been granted permission to conduct these fields and materials, and we have followed national and local regulations. We ensured the collection of rice experimental materials has no negative consequences for the local environment.

## Supplementary Information

Supplementary Information.
