# Supplementary material for: Exploring japonica rice epigenetic diversity in the main production regions of Heilongjiang Province
Source: Sci Rep. 2022 Mar 17;12:4592. doi: 10.1038/s41598-022-08683-2 (PMC8931079; doi:10.1038/s41598-022-08683-2)
Supplement: Supplementary file 1 — Supplementary Information. [file 41598_2022_8683_MOESM1_ESM.docx]

**
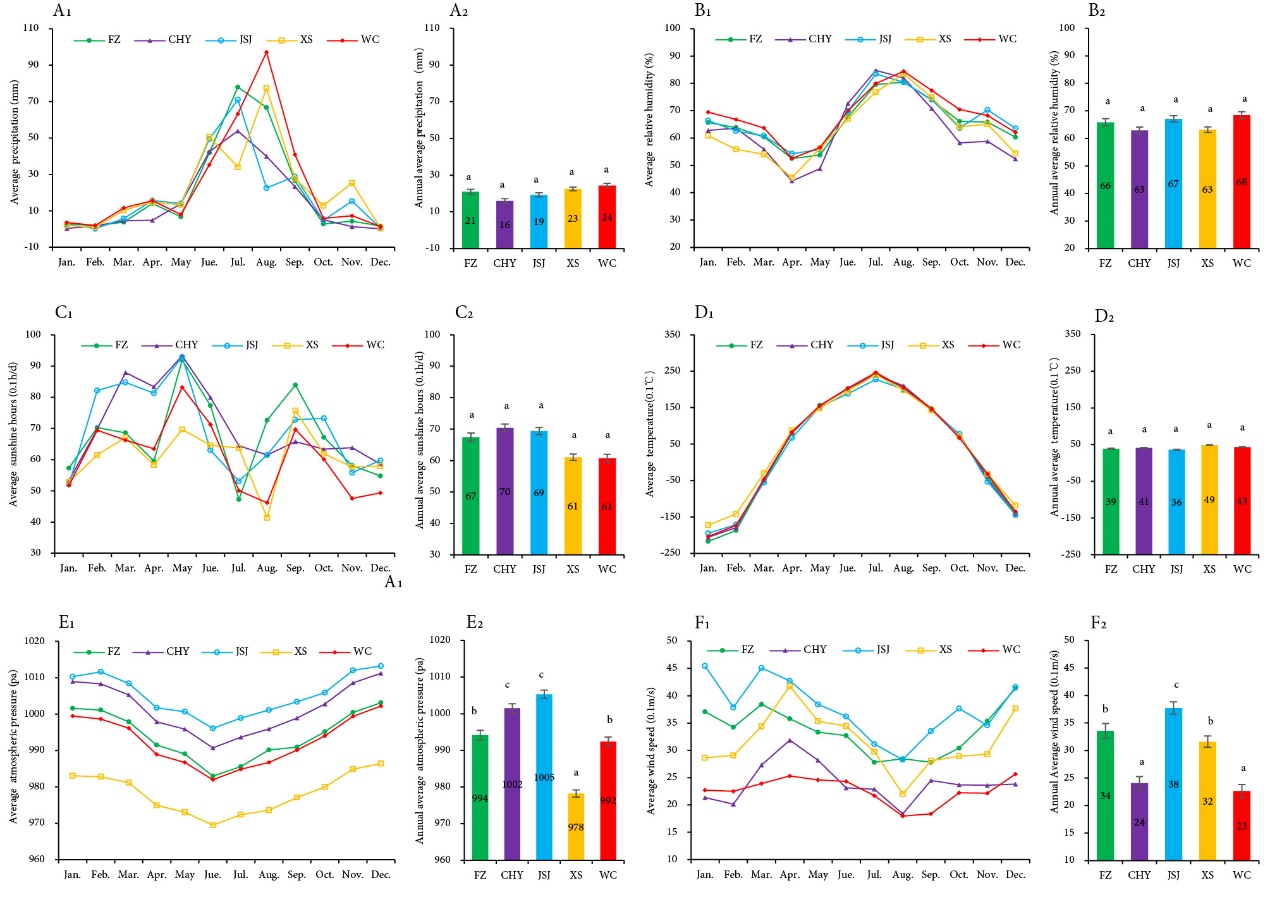
**

**Figure S1.** Line and bar charts depicting the general environmental conditions at the rice sample collection sites in Heilongjiang Province. A_1_. Monthly average precipitation; A_2_. Annual average precipitation for each region; B_1._ Monthly average relative humidity; B_2_. Annual average relative humidity for each region; C_1_. Monthly average sunshine hours; C_2_. Annual average sunshine hours for each region; D_1_. Monthly average temperature; D_2_. Annual average temperature for each region; E_1_. Monthly average atmospheric pressure; E_2_. Annual average atmospheric pressure for each region; F_1_. Monthly average wind speed; F_2_. Annual average wind speed for each region. Significant differences between groups are denoted by different letters (p<0.05).

| **Region** | **1000-grain weight**  **(g)** | **Plant height**  **(cm)** | **Brown rice length**  **(mm)** | **Brown rice shape**  **(Length-width ratio)** | **Panicle length (cm)** |
| --- | --- | --- | --- | --- | --- |
| FZ | 25.76±1.62 ^a^ | 91.3±2.7^ab^ | 5.8±0.2^b^ | 2.3±0.14 ^b^ | 17.5±1.2 ^b^ |
| CHY | 26.85±1.37 ^ab^ | 89.8±2.2^a^ | 5.2±0.2 ^a^ | 1.8±0.06 ^a^ | 16.5±1.6 ^ab^ |
| JSJ | 25.73±1.35 ^a^ | 95.0±2.4^bc^ | 5.1±0.2 ^a^ | 1.7±0.05 ^a^ | 16.2±1.0 ^a^ |
| XS | 26.89±0.85 ^b^ | 98.6±1.9 ^c^ | 6.5±0.3 ^c^ | 2.6±0.09 ^c^ | 19.9±1.8 ^c^ |
| WC | 27.09±0.79 ^b^ | 99.5±2.0 ^c^ | 6.5±0.2 ^c^ | 2.6±0.08 ^c^ | 20.7±1.3 ^c^ |

**Table S1.** Phenotypic diversity of rice in different regions. Significant differences between groups are denoted by different letters (p<0.05).
